# Primary Spinal Germ Cell Tumors: A Case Analysis and Review of Treatment Paradigms

**DOI:** 10.1155/2013/798358

**Published:** 2013-11-07

**Authors:** Joshua J. Loya, Henry Jung, Caroline Temmins, Nam Cho, Harminder Singh

**Affiliations:** ^1^Department of Neurosurgery, Stanford University School of Medicine, Edwards Building, Neurosurgery Mailroom, 300 Pasteur Drive, Stanford, CA 94305-5487, USA; ^2^Department of Pathology, Santa Clara Valley Medical Center (SCVMC), 751 South Bascom Ave., San Jose, CA 95128, USA; ^3^Department of Radiation Oncology, Santa Clara Valley Medical Center (SCVMC), 751 South Bascom Ave., San Jose, CA 95128, USA

## Abstract

*Objective*. Primary intramedullary spinal germ cell tumors are exceedingly rare. As such, there are no established treatment paradigms. We describe our management for spinal germ cell tumors and a review of the literature. *Clinical Presentation*. We describe the case of a 45-year-old man with progressive lower extremity weakness and sensory deficits. He was found to have enhancing intramedullary mass lesions in the thoracic spinal cord, and pathology was consistent with an intramedullary germ cell tumor. A video presentation of the case and surgical approach is provided. *Conclusion*. As spinal cord germinomas are highly sensitive to radiation and chemotherapy, a patient can be spared radical surgery. Diverse treatment approaches exist across institutions. We advocate biopsy followed by local radiation, with or without adjuvant chemotherapy, as the optimal treatment for these tumors. Histological findings have prognostic value if syncytiotrophoblastic giant cells (STGCs) are found, which are associated with a higher rate of recurrence. The recurrence rate in STGC-positive spinal germinomas is 33% (2/6), whereas it is only 8% in STGC-negative tumors (2/24). We advocate limited volume radiotherapy combined with systemic chemotherapy in patients with high risk of recurrence. To reduce endocrine and neurocognitive side effects, cranio-spinal radiation should be used as a last resort in patients with recurrence.

## 1. Introduction

Germ cell tumors are similar in histology to germinal cells of the genital organs, and they may aberrantly arise in the central nervous system (CNS). Germ cell tumors account for 1% of all CNS tumors, and they are seen more frequently in Japan (3%) and East Asia (12.5%) [1]. They usually occur in the pineal or suprasellar regions and less frequently in the thalamus, basal ganglia, or ventricles. Spinal seeding of a germ cell tumor has been well documented in the form of drop metastases [2–4]; however, extremely rarely, germ cell tumors may be found within the spinal cord as a primary tumor. 

We present a patient with a primary intramedullary thoracic germ cell tumor. This patient is one of only a handful of reports of a primary spinal germ cell tumor from the western hemisphere (Table 1).

## 2. Case Report

### 2.1. Presentation

A 45-year-old man presented with numbness and weakness in his lower extremities, worsening over the course of a year. His weakness substantially worsened over the 4 months prior to presentation, during which he became fully dependent on a walker. He was also intermittently incontinent of urine and chronically constipated.

On physical examination, from the T2 level and below, he had right loss greater than left loss of light touch, pinprick, and proprioception sensation. On motor examination, his right leg strength was 0/5 and his left leg strength was 4/5. He had bilateral clonus and muscle spasms in his legs. 

### 2.2. Imaging

**Figure 1:**
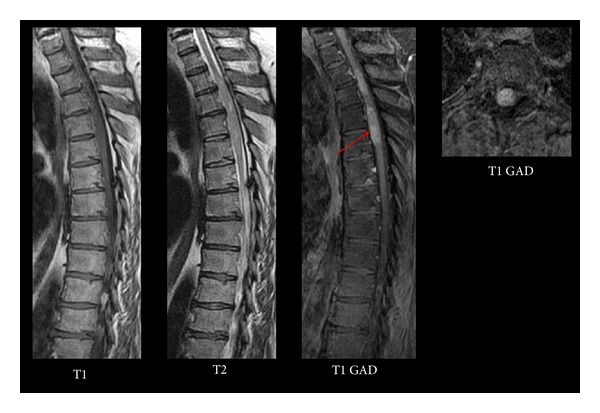
Preoperative thoracic MRI shows three sagittal sequences (T1, T2, and T1 with gadolinium) on the left and an axial (T1 with gadolinium) on the right demonstrating an intramedullary mass between T2 and T5 (arrow).

Magnetic resonance imaging (MRI) demonstrated intramedullary signal within the upper thoracic spinal cord with diffuse enhancement and cord expansion (Figure 1). Imaging of the remaining neural axis was negative. Testicular ultrasound and a CT of chest, abdomen, and pelvis were normal. Serum and CSF levels of AFP and *β*-HCG were within normal limits. 

Differential diagnosis of this lesion included primary spinal cord neoplasm, such as astrocytoma and ependymoma, hemangioblastoma, intradural metastasis, and lymphoma. Ischemia and lupus myelitis were also included in the differential diagnosis. The patient was taken for open biopsy/resection of this lesion. 

### 2.3. Operation

The patient was placed prone on the operating room table. Motor and sensory neuromonitoring was established prior to incision. Motor evoked potentials (MEPs) and somatosensory evoked potentials (SSEPs) were absent below the level of the lesion. The correct levels were ascertained using fluoroscopy. Bilateral laminectomies were then performed at T3, T4, and T5. After the thecal sac was decompressed, attention was turned towards opening the dura and identifying the tumor. At this time, the microscope was brought into the operation. Under the microscope, the dura was opened. The cord was noted to have yellow discoloration, but otherwise no gross abnormality was observed (see supplemental video in Supplementary Material available online at http://dx.doi.org/10.1155/2013/798358, which demonstrates surgical approach and technique). 

The posterior median sulcus was then identified, and a midline myelotomy was performed. Upon entering the cord, the abnormal tissue appeared grayish and invasive and displayed no clear border between tumor and normal tissue. A biopsy sample was sent to pathology. An intraoperative frozen section favored a malignant metastasis or high-grade tumor. Given its lack of clear boundaries, poor neuromonitoring signals, and the risk of further compromise of his remaining neurological function, aggressive tumor debulking was not pursued. 

### 2.4. Pathology

**Figure 2:**
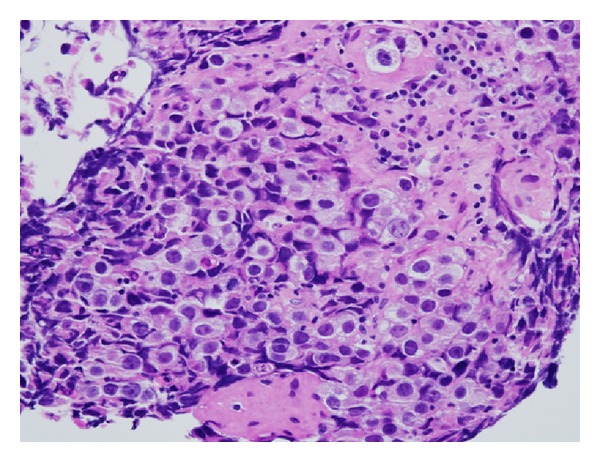
Two-cell pattern of germ cell tumors (hematoxylin and eosin stain).

Pathology demonstrated the typical two-cell pattern of germinomas (Figure 2). There was an infiltrate of cells with large prominent nuclei with well-defined borders and clear cytoplasm (see supplemental video for pathology images). There were also small round lymphocytes infiltrating the stroma. Immunohistologic staining was positive for placental alkaline phosphatase (PLAP), OCT3/4, and CD117. That, along with the dot-like positivity of the cytokeratin AE1/AE3, supported the diagnosis of a germ cell tumor. No syncytiotrophoblastic giant cells (STGCs) were present. 

### 2.5. Postoperative Course

**Figure 3:**
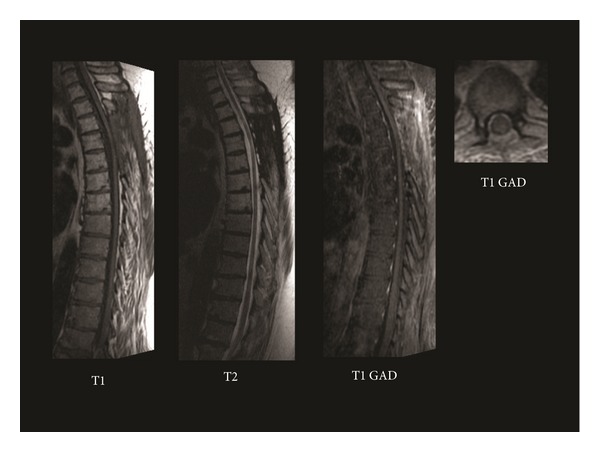
Postoperative MRI at 1-year followup shows 3 sagittal sequences (T1, T2, and T1 with gadolinium) on the left and an axial (T1 with gadolinium) on the right demonstrating no evidence of tumor recurrence.

This patient received standard, fractionated external beam radiation therapy totaling 45 Gy in 1.8 Gy fractions to the upper thoracic spine. The patient's neurological symptoms remained stable throughout his treatment course with mild improvement of the right lower extremity strength. Repeat imaging at 4 months showed complete resolution of the intramedullary spinal cord lesion. He received no chemotherapy due to his excellent response to radiation treatment. At 1-year followup, he remained free of radiographic evidence of disease with no drop metastases (Figure 3), and at 23-month followup, he remained stable on clinical evaluation.

## 3. Discussion and Review of the Literature

We found 29 reported cases of primary spinal germ cell tumors in the literature, most having been described in young Japanese adults (22/29) (Table 1). Spinal germinomas appear to occur slightly more often in males than in females (60% males versus 40% females). Similarly, pineal germinomas have a significantly higher incidence in males than in females [31]. Like intracranial germinomas, spinal germinomas occur primarily in the young population. We found the mean age to be approximately 26 years old with a median age of 28 years, ranging from 5 to 45 years. Our 45-year-old patient was the oldest of the cohort.

Spinal germinomas occurred most frequently within the thoracic spinal cord (47%), followed by the thoracolumbar area (27%), the lumbar area (20%), and least frequently the cervical spinal cord (7%). Spinal germinomas are also primarily seen intramedullary in 70% of cases. They had both intramedullary and extramedullary components in 17% of cases and were purely extramedullary in 13% of cases. 

Histological findings of CNS germinomas may have prognostic value if STGCs are found, as these are associated with poorer prognosis and a higher rate of recurrence [32]. While STGCs characteristically produce *β*-HCG, which may lead to CSF or serum elevation of this hormone, elevated *β*-HCG levels do not definitively indicate the presence of STGCs. 

We found that while 37% (11/30) of cases had elevated levels of *β*-HCG, only 55% (6/11) of these 11 were STGC positive. However, 100% of cases that were STGC positive were also *β*-HCG positive (6/6) (Table 1).

The overall recurrence rate of spinal cord germinomas after treatment was 13% (4/30), bolded in Table 1. The recurrence rate in STGC-positive germinomas was 33% (2/6), whereas the recurrence rate was only 8% (2/24) in STGC-negative tumors (Table 1).

Clinical symptoms and imaging features of primary spinal germ cell tumors are often indistinguishable from other spinal cord neoplasms. Only histological examination can establish a definitive diagnosis. If frozen-section biopsy during surgery suggests the possible diagnosis of germinoma, radical resection of the tumor should not be undertaken. However, if frozen section favors a high-grade tumor (as in our case), the extent of resection must be guided by preoperative neurological function, the reliability of neuromonitoring signals, the extent of cord compression, and the amount of medullary invasion.

Like intracranial germinomas, spinal cord germinomas are also highly sensitive to radiation. Most cases can be successfully treated with external beam radiotherapy despite the lack of standard guidelines for fractionation and dose. In our particular case, 3D conformal external beam radiation therapy (EBRT) was utilized to treat the spinal cord from C7 to T7. This field was treated to a dose of 45 Gy in 25 fractions using 15 MV photons and AP/PA portals. Daily kV imaging was performed for image-guided radiotherapy (IGRT) to ensure precise patient positioning. In our review, we found the average amount of local radiation to be 35.7 ± 13.4 Gy and the average amount of craniospinal radiation to be 30.4 ± 12.1 Gy. In order to reduce the endocrine and neurocognitive side effects of craniospinal radiation, it should be reserved for patients with STGC-positive tumors or patients with recurrence. 

Intracranial germ cell tumors are also highly sensitive to chemotherapy, especially to platinum and alkylating agents. However, there are no standard guidelines for chemotherapy for spinal germinomas. In our case review, 50% of the patients received systemic chemotherapy. 

The common chemotherapeutic agents found to be effective for CNS germinomas are cisplatin, bleomycin, vinblastine, and etoposide. The treatment with chemotherapy alone in CNS germinomas has been investigated [33]. In one study, 37 of 45 patients (82%) had a complete response to chemotherapy, but 20 of these patients relapsed at 18 months (median time) from diagnosis. Fortunately, most of these patients were salvaged with further therapy, which included radiation. Other reports corroborate that chemotherapy alone may have increased rates of relapse with subsequent need for salvage radiation [34].

One report describes the complete remission of a pineal gland germinoma after 2 cycles of BEP therapy (bleomycin, etoposide, and cisplatin) with no further need for radiotherapy [35]. However, the patient of this report was only followed up for 15 months following tumor eradication. Others have reported great success in treating solitary pure germinomas with 3 or 4 cycles of cisplatin and etoposide (EP regimen), followed by 24 Gy local radiation therapy, as well as in treating HCG-secreting, multifocal, or disseminated germinomas with 4 to 5 cycles of ifosfamide, cisplatin, and etoposide (ICE regimen), followed by 24 Gy local radiation therapy [36]. Thus, the EP and ICE regimens can be highly effective in treating CNS germinomas and can reduce the dose and volume of radiotherapy. 

Generally, some form of radiation is recommended to maintain long-term control. Although chemotherapy has the advantage of decreasing the total dose of radiation, chemotherapy can have significant side effects. A treatment regimen consisting of PVB therapy (cisplatin, vinblastine, and bleomycin) or BEP therapy avoids the use of alkylating agents and can spare sterility; yet it can have significant renal and auditory toxicity [37]. Other long-term complications of chemotherapy have been recognized, such as secondary leukemia and solid tumors. 

## 4. Conclusion

Although primary spinal cord germinomas are extremely rare, there are reports of these tumors primarily in the Japanese literature. Definitive diagnosis is imperative. As spinal cord germinomas are highly sensitive to radiation and chemotherapy, a patient can be spared radical surgery. Recurrence of spinal germinomas is uncommon and associated with STGC positivity. 

We advocate surgical biopsy followed by local radiation, with or without adjuvant chemotherapy, as the optimal means of treatment of spinal germ cell tumors with low risk of recurrence. Taking cue from the management of intracranial germ cell tumors, limited volume radiotherapy combined with systemic chemotherapy should be offered in patients with high risk of recurrence, for example, those with high serum/CSF B-HCG levels and intratumoral presence of STGCs.

Craniospinal irradiation should be used as a last resort (due to its many neuroendocrine and neurocognitive side effects) in patients with recurrence.

## Supplementary Material

Supplementary video consists of a complete narrated presentation of the clinical features, diagnostic considerations, imaging, surgical approach, pathology, and follow-up evaluations of the case presented. It includes intraoperative footage of surgical technique from positioning to tumor resection and closure. It also includes detailed pathological images with appropriate histological stains used in tissue evaluation and diagnosis. Finally it concludes with postoperative imaging and clinical status at long term follow-up.

## Figures and Tables

**Table 1:** Reported cases of primary spinal germ cell tumors.

Patient no.	Series	Country	Age/sex	Location	Medullary	Operation	Radiation craniospinal (Gy)	Radiation Local (Gy)	Chemotherapy	HCG	STGC	Followup	Recurrence
**1**	**Hisa et al. [5]**	**Japan**	**5/M**	**T11-L3**	**IM, EM**	**Bx**	**—**	**35 + 75**	**Actinomycin + methotrexate + vincristine/bleomycin + cisplatin + vinblastine**	**+**	**+**	**6 months**	**R, NR after amputation of spinal cord**
2	Zhu et al. [6]	China	7/M	T12-L1	IM	PR	ND	ND	—	**+**	**+**	—	—
3	Slagel et al. [7]	Japan	16/F	T11-L4	IM, EM	PR	—	30	—	—	—	28 months	NR
4	Aoyama et al. [8]	Japan	16/F	T9-T12	IM	PR	—	30.6	Ifosfamide + cisplatin + etoposide	**+**	—	3 years	NR
5	Chute et al. [9]	US	18/M	T6-T8	IM	Bx	—	50	—	**+**	—	6 months	NR
6	Huang et al. [10]	US	18/M	C3-C6	IM	PR	ND	ND	Bleomycin + cisplatin + etoposide	—	—	6 months	NR
7	Kiyuna et al. [11]	Japan	20/F	T11-L3	EM	TR	30.4	40	—	—	—	2 years	NR
8	Massimino et al. [12]	Italy	20/M	L2	IM	TR	30	9	Cisplatin + etoposide + bleomycin	**+**	**+**	33 months	NR
9	Kinoshita et al. [13]	Japan	21/F	T9-T11, L2-L3	IM	PR	—	25.2	Carboplatin + etoposide	—	—	3 years	NR
10	Takahashi et al. [14]	Japan	22/F	L1-L2	IM, EM	PR	57	30.6	Ifosfamide + carboplatin or cisplatin + etoposide	**+**	—	1.5 years	NR
11	Yamagata et al. [15]	Japan	24/F	T6-T7	IM	PR	25.6	19.8	Ifosfamide + cisplatin + etoposide	**+**	**+**	6 months	NR
12	Kawano and Tsujimura [16]	Japan	24/M	L1-L3	IM, EM	PR	16	40	—	—	—	ND	NR
13	Itoh et al. [17]	Japan	24/M	T11-T12	IM	TR	24.1	52.1	—	—	—	13 months	NR
14	Miyauchi et al. [18]	Japan	24/M	T12-L3	IM, EM	PR	15	40	—	—	—	15 months	NR
**15**	**Nakata et al. [19]**	**Japan**	**27/M**	**T7-T9**	**IM**	**PR**	**—**	**48**	**—**	**—**	**—**	**1 year**	**R**
**16**	**Biswas et al. [20]**	**India**	**28/M**	**L2-L4**	**EM**	**TR**	**—**	**20**	**Bleomycin + cisplatin + etoposide**	**+**	**+**	**11 months**	**R**
**17**	**Tekkök and Sav** **[21]**	**Turkey**	**28/M**	**L1-S2**	**EM**	**TR**	**54**	**51**	**Cisplatin + etoposide + bleomycin**	**—**	**—**	**22 months**	**R, NR after chemotherapy and reresection**
18	Ganslandt et al. [22]	Germany	29/M	T12-L4	IM	TR	32	18.4	—	—	—	2 years	NR
19	Matsuoka et al. [23]	Japan	31/F	T12-L2	IM	PR	—	50	—	—	—	15 months	NR
20	Nagasawa et al. [24]	Japan	31/M	Midcervical	IM	—	—	51	—	—	—	40 months	NR
21	Sasaki et al. [25]	Japan	32/F	T3-T4	IM	—	27.5	19.8	Methotrexate + etoposide	**+**	**+**	5 years	NR
22	Watanabe et al. [26]	Japan	33/F	T1-T3	IM	PR	—	30	Carboplatin + etoposide	ND	—	1 year	NR
23	Hata et al. [27]	Japan	33/M	T7-T9	IM	PR	—	36	Carboplatin + etoposide	—	—	38 months	NR
24	Yamagata et al. [15]	Japan	33/M	T9-T11	IM	PR	24	—	Cisplatin + etoposide	—	—	2 years	NR
25	Hanafusa et al. [28]	Japan	34/F	T10-T11	IM	TR	30	45	—	—	—	24 months	NR
26	Matsuyama et al. [29]	Japan	34/F	T6-T8	IM	PR	—	46	—	—	—	12 months	NR
27	Aoyama et al. [8]	Japan	34/F	T8-T10	IM	Bx	—	30.6	Ifosfamide + cisplatin + etoposide	**+**	—	2 years	NR
28	Nakata et al. [19]	Japan	35/M	T6-T7	IM	TR	24	—	Carboplatin + etoposide	**+**	—	2 years	NR
29	Horvath et al. [30]	Japan	43/M	L1-L2	EM	TR	36	14	—	—	—	8 months	NR
30	Present case	USA	45/M	T2-T5	IM	PR	—	45	—	—	—	22 months	NR

Bx: biopsy; PR: partial resection; TR: total resection; HCG: *β*-human chorionic gonadotropin; STGC: syncytiotrophoblastic giant cells; M: male; F: female; R: recurrence; NR: norecurrence; bold font: patients with recurrence; IM: intramedullary; EM: extramedullary.

## References

[1] Borg M (2003). Germ cell tumours of the central nervous system in children—controversies in radiotherapy. *Medical and Pediatric Oncology*.

[2] Hanakita S, Takenobu A, Kambe A, Watanabe T, Shin M, Teraoka A (2012). Intramedullary recurrence of germinoma in the spinal cord 15 years after complete remission of a pineal lesion: case report. *Journal of Neurosurgery*.

[3] Kahn L, Fridley J, Patel AJ (2012). Disseminated germinoma in the brain and cervical spinal cord 10 years after radiographic resolution of pineal germinoma. *Journal of Clinical Neuroscience*.

[4] Ogawa K, Yoshii Y, Shikama N (2008). Spinal recurrence from intracranial germinoma: risk factors and treatment outcome for spinal recurrence. *International Journal of Radiation Oncology Biology Physics*.

[5] Hisa S, Morinaga S, Kobayashi Y (1985). Intramedullary spinal cord germinoma producing HCG and precocious puberty in a boy. *Cancer*.

[6] Zhu J, Gao Y, Zheng W, Yang J (2002). Intramedullary spinal cord germinoma: a case report. *Chinese Medical Journal*.

[7] Slagel DD, Goeken JA, Platz CA, Moore SA (1995). Primary germinoma of the spinal cord: a case report with 28-year follow-up and review of the literature. *Acta Neuropathologica*.

[8] Aoyama T, Hida K, Ishii N, Seki T, Ikeda J, Iwasaki Y (2007). Intramedullary spinal cord germinoma-2 case reports. *Surgical Neurology*.

[9] Chute DJ, Burton EC, Klement IA, Frazee JG, Vinters HV (2003). Primary intramedullary spinal cord germinoma: case report. *Journal of Neuro-Oncology*.

[10] Huang JH, Tsui I, Judkins AR, Simon E, Birknes JK, Sutton LN (2004). Intramedullary cervical spine germinoma: case report. *Neurosurgery*.

[11] Kiyuna M, Toda T, Sadi AM, Toyoda Z, Nakashima Y (1999). A rare case of extramedullary spinal cord germinoma. *Pathology International*.

[12] Massimino M, Gandola L, Spreafico F (2006). Unusual primary secreting germ cell tumor of the spine: case report. *Journal of Neurosurgery*.

[13] Kinoshita Y, Akatsuka K, Ohtake M, Kamitani H, Watanabe T (2010). Primary intramedullary spinal cord germinoma. *Neurologia Medico-Chirurgica*.

[14] Takahashi M, Koyama H, Matsubara T, Murata H, Miura K, Nagano A (2006). Mixed germinoma and choriocarcinoma in the intramedullary spinal cord: case report and review of the literature. *Journal of Neuro-Oncology*.

[15] Yamagata T, Takami T, Tsuyuguchi N, Goto T, Wakasa K, Ohata K (2009). Primary intramedullary spinal cord germinoma: diagnostic challenge and treatment strategy—two case reports. *Neurologia Medico-Chirurgica*.

[16] Kawano K, Tsujimura T (1995). Intramedullary spinal cord germinoma. *Arch Histopathol Differ Diagn*.

[17] Itoh Y, Mineura K, Sasajima H, Kowada M (1996). Intramedullary spinal cord germinoma: case report and review of the literature. *Neurosurgery*.

[18] Miyauchi A, Matsumoto K, Kohmura E, Doi T, Hashimoto K, Kawano K (1996). Primary intramedullary spinal cord germinoma: case report. *Journal of Neurosurgery*.

[19] Nakata Y, Yagishita A, Arai N (2006). Two patients with intraspinal germinoma associated with Klinefelter syndrome: case report and review of the literature. *American Journal of Neuroradiology*.

[20] Biswas A, Puri T, Goyal S (2009). Spinal intradural primary germ cell tumour-review of literature and case report. *Acta Neurochirurgica*.

[21] Tekkök IH, Sav A (2005). Aggressive spinal germinoma with ascending metastases. *Journal of Neuro-Oncology*.

[22] Ganslandt O, Buchfelder M, Grabenbauer GG (2000). Primary spinal germinoma in a patient with concomitant Klinefelter’s syndrome. *British Journal of Neurosurgery*.

[23] Matsuoka S, Itoh M, Shinonome T, Tanimura A (1991). Intramedullary spinal cord germinoma: case report. *Surgical Neurology*.

[24] Nagasawa S, Kikuchi H, Yamashita J, Mitsuno K (1991). Intracranial and spinal germinomas occurring four years after spinal cord germinoma. Case report. *Neurologia Medico-Chirurgica*.

[25] Sasaki T, Amano T, Takao M, Shibata M, Shigematsu N, Fukuuchi Y (2002). A case of intramedullary spinal cord tumor producing human chorionic gonadotropin. *Journal of Neuro-Oncology*.

[26] Watanabe A, Horikoshi T, Naganuma H, Satoh E, Nukui H (2005). Intramedullary spinal cord germinoma expresses the protooncogene c-kit. *Acta Neurochirurgica*.

[27] Hata M, Ogino I, Sakata K, Murata H, Kawano N, Matsubara S (2002). Intramedullary spinal cord germinoma: case report and review of the literature. *Radiology*.

[28] Hanafusa K, Shibuya H, Abe M, Yamaura K, Suzuki S (1993). Intramedullary spinal cord germinoma. Case report and review of the literature. *RoFo Fortschritte auf dem Gebiete der Rontgenstrahlen und der Neuen Bildgebenden Verfahren*.

[29] Matsuyama Y, Nagasaka T, Mimatsu K, Inoue K, Mii K, Iwata H (1995). Intramedullary spinal cord germinoma. *Spine*.

[30] Horvath L, McDowell D, Stevens G, Parkinson R, McCarthy S, Boyer M (2001). Case 2. Seminoma of the conus medullaris. *Journal of Clinical Oncology*.

[31] Goodwin TL, Sainani K, Fisher PG (2009). Incidence patterns of central nervous system germ cell tumors: a SEER study. *Journal of Pediatric Hematology/Oncology*.

[32] Uematsu Y, Tsuura Y, Miyamoto K, Itakura T, Hayashi S, Komai N (1992). The recurrence of primary intracranial germinomas. Special reference to germinoma with STGC (syncytiotrophoblastic giant cell). *Journal of Neuro-Oncology*.

[33] Balmaceda C, Heller G, Rosenblum M (1996). Chemotherapy without irradiation—a novel approach for newly diagnosed CNS germ cell tumors: results of an international cooperative trial. *Journal of Clinical Oncology*.

[34] Itoyama Y, Kochi M, Yamashiro S, Yoshizato K, Kuratsu J, Ushio Y (1993). Combination chemotherapy with cisplatin and etoposide for hematogenous spinal metastasis of intracranial germinoma. Case report. *Neurologia Medico-Chirurgica*.

[35] Hupperets PSGJ, Defesche HF, de Bruijckere LM, Twijnstra A (1999). The role of chemotherapy in intracranial germinoma: a case report. *Annals of Oncology*.

[36] Sawamura Y, Shirato H, Ikeda J (1998). Induction chemotherapy followed by reduced-volume radiation therapy for newly diagnosed central nervous system germinoma. *Journal of Neurosurgery*.

[37] Pinkerton CR, Broadbent V, Horwich A (1990). ‘JEB’—a carboplatin based regimen for malignant germ cell tumours in children. *British Journal of Cancer*.

